# DFT study of the effect of substituents on the absorption and emission spectra of Indigo

**DOI:** 10.1186/1752-153X-6-70

**Published:** 2012-07-18

**Authors:** Francisco Cervantes-Navarro, Daniel Glossman-Mitnik

**Affiliations:** 1 NANOCOSMOS Virtual Lab, Centro de Investigación en Materiales Avanzados, Miguel de Cervantes 120, Complejo Industrial Chihuahua, Chihuahua, Chih 31109, Mexico

**Keywords:** Indigo, Absorption spectra, Emission spectra, Effect of substituents, DFT, CIS

## Abstract

**Background:**

Theoretical analyses of the indigo dye molecule and its derivatives with Chlorine (Cl), Sulfur (S), Selenium (Se) and Bromine (Br) substituents, as well as an analysis of the Hemi-Indigo molecule, were performed using the Gaussian 03 software package.

**Results:**

Calculations were performed based on the framework of density functional theory (DFT) with the Becke 3- parameter-Lee-Yang-Parr (B3LYP) functional, where the 6-31 G(d,p) basis set was employed. The configuration interaction singles (CIS) method with the same basis set was employed for the analysis of excited states and for the acquisition of the emission spectra.

**Conclusions:**

The presented absorption and emission spectra were affected by the substitution position. When a hydrogen atom of the molecule was substituted by Cl or Br, practically no change in the absorbed and emitted energies relative to those of the indigo molecule were observed; however, when N was substituted by S or Se, the absorbed and emitted energies increased.

## Introduction

Substituent effects on molecules have always been a subject of study because it is our goal to modify molecules based on our needs. A way in which to study this phenomenon is to analyze the effects of substituents on the spectra of molecules. Solvent [1], substituent [2] and synthesis effects [3], as well as combinations of these effects [4], have been shown.

In the present study, the indigo molecule was employed as the base molecule, with Chlorine (Cl), Bromine (Br), Sulfur (S) and Selenium (Se) as substituents [5]. The Hemi-Indigo molecule [6,7] was also studied because its properties have been shown to be similar to those of the Indigo molecule; we also thought it would be interesting to compare its properties with those of the rest of the investigated molecules.

Theoretical studies of the effects of substituents on absorption and emission spectra [8-16] have been performed, including studies on the indigo molecule [17]. The present work attempts to explain, perhaps vaguely but completely based on the obtained results, the effects observed when the absorption and emission spectra of indigo are compared.

## Theory and computational details

GAUSSVIEW 03 software was used to generate the molecular structures, and calculations were performed using GAUSSIAN 03 W. Density functional theory (DFT) was implemented for the frequency and energy optimization. Time-dependent DFT (TD-DFT) and the configuration interaction singles (CIS) method [18] were employed for the theoretical study of excited states. For all the study was employed the same level of theory, the 6-31 G(d,p) basis set and the Becke 3-parameter-Lee-Yang-Parr (B3LYP) functional were employed, according to the work of Perpète and Jacquemin (2009)[19]; the basis set was using because we calculate the molecule of indigo in gas phase with both basis set 6-31 G(d,p) and 6-311 G(2d,p) and the transition from the first one is the most similar to experimental value 540 nm [20] (536.2 nm with 6-31 G(d,p) and 550.4 nm with 6-3111(2d,p)).

**Figure 1:**
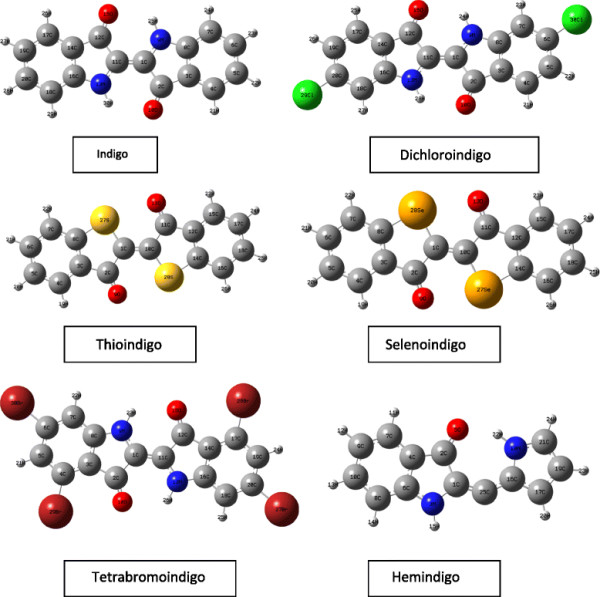
Optimized molecular structures of the molecules under study.

Figure 1 shows the optimized structure of the studied molecules. TD-DFT was used to obtain the absorption spectra of the first six excited states. To obtain the emission spectra, the first dominant excited state was optimized with CIS calculations, and energy calculations were then performed using TD-DFT for the first six excited states.

The absorption and emission spectral data were processed using the SWizard software program [21], and the data were then plotted and compared in a spreadsheet. In addition, the assignments of the transitions observed in the calculations were performed.

**Figure 2:**
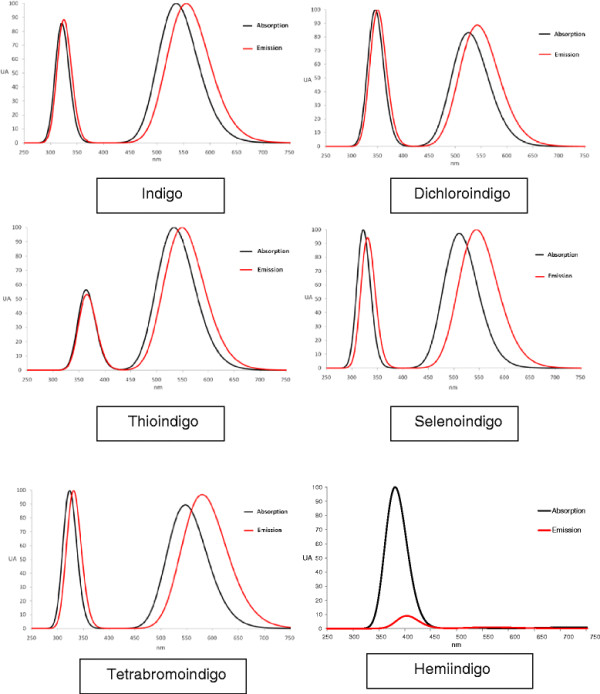
**Absorption and emission spectra of the molecules under study.** The intensity is in arbitrary units but relative to the highest intensity for each molecule.

Finally, the absorption and emission spectra were overlapped for each molecule, and the energies of the corresponding transitions were compared Figure 2.

## Results and discussions

In Table 1, we show a summary of the calculated results; the transition wavelengths, their equivalent values in eV, the oscillator force and the assignments of the corresponding transitions are listed for each molecule.

**Table 1:** Calculation results for the absorption and emission of each molecule

		**nm**	**eV**	**(f)**	**Assignment**
Indigo	Absorption	536.2	2.31	0.2673	S H-0 → L + 0
		321.2	3.86	0.2291	S H-3 → L + 0
	Emission	555.3	2.23	0.2771	S H-0 → L + 0
		325.2	3.81	0.245	S H-3 → L + 0
Dichloroindigo	Absorption	525.4	2.36	0.2973	S H-0 → L + 0
		345.3	3.59	0.3578	S H-3 → L + 0
	Emission	542.7	2.28	0.3139	S H-0 → L + 0
		350	3.54	0.3544	S H-2 → L + 0
Selenoindigo	Absorption	547.4	2.26	0.1697	S H-0 → L + 0
		323.4	3.83	0.1897	S H-4 → L + 0
	Emission	580.1	2.14	0.1889	S H-0 → L + 0
		331.1	3.74	0.195	S H-4 → L + 0
Thioindigo	Absorption	510.7	2.43	0.1964	S H-0 → L + 0
		321.8	3.85	0.202	S H-3 → L + 0
	Emission	544.6	2.28	0.2191	S H-0 → L + 0
		330.3	3.75	0.2063	S H-4 → L + 0
Tetrabromoindigo	Absorption	533.4	2.32	0.3934	S H-0 → L + 0
		363.6	3.41	0.2215	S H-2 → L + 0
	Emission	549.1	2.26	0.419	S H-0 → L + 0
		365.1	3.4	0.2226	S H-2 → L + 0
Hemiindigo	Absorption	394.1	3.15	0.0551	S H-1 → L + 0
		375.6	3.3	0.0995	S H-2 → L + −1
	Emission	629.1	1.97	0.0002	S H-0 → L + 0 H-1 → L + −1
		403	3.08	0.0122	S H-1 → L + 0 H-2 → L + −1

Only the main transitions, based on their oscillator force, are shown in Table 1. For Indigo, the HOMO-0 to LUMO-0 transition appeared to be the most likely; however, a transition from a lower-level HOMO-3 to LUMO-0 is allowed due to the high energies at which it also absorbs. In the same way, for its emission spectrum or transitions, the most likely transition is from LUMO-0 to HOMO-0. This result is in accordance with the fact that promoted electrons have less energy than electrons coming from HOMO-3; therefore, the last electrons return to their original state from LUMO-0 to HOMO-3.

The observed Indigo and Tetrabromoindigo behaviors are similar to those of the other molecules. The HOMO-2 to LUMO-0 (and vice-versa) transition probabilities are approximately the same; however, the HOMO-0 to LUMO-0 (and vice-versa) transitions exhibit strongly increased transition probabilities.

Dichloroindigo and Selenoindigo show similar behaviors in that the HOMO-0 to LUMO-0 (and vice-versa) transitions are not the most likely transitions. Nevertheless, they are different because Dichloroindigo exhibits an increased transition probability compared to Indigo, whereas Selenoindigo exhibits a decreased probability.

Dichloroindigo shows absorption from the HOMO-3 to LUMO-0 transition but exhibits a different emission transition from LUMO-0 to HOMO-2. Therefore, HOMO-2 electrons can be assumed to transition to HOMO-3 before the LUMO-0 to HOMO-2 transition occurs. This transition may be because HOMO-3 and HOMO-2 exhibit similar energies.

Differences were found between Thioindigo and Indigo. First, the transition probabilities are lower for Thioindigo, and second, the most likely absorption transition is the HOMO-3 to LUMO-0 transition, followed by the HOMO-0 to LUMO-0 transition. However, the most likely emission transition is LUMO-0 to HOMO-0, followed by the LUMO-0 to HOMO-4 transition. This result could be due to the fact that HOMO-3 and HOMO-4 exhibit similar energies, such that after some electrons are promoted from HOMO-3, HOMO-4 electrons replace them. Therefore, electrons promoted from HOMO-3, after losing their energy, return to HOMO-4.

For Dichloroindigo, the HOMO-2 energy must be slightly higher than that of HOMO-3; this situation differs from that of Thioindigo, where for the transition to occur at a lower energy level, the molecule must, in theory, have the same HOMO-3 and HOMO-4 energy levels.

Hemiindigo transitions and transitions from HOMOs lower than HOMO-0 are not likely. Notably, in the case of Hemi-Indigo, absorptions and transitions occur at different wavelengths for each transition, namely HOMO-1 to LUMO-0 at 394.1 nm and HOMO-1 to LUMO-1 at 375.6 nm. However, emission occurs at the same wavelength, namely 403 nm, for the LUMO-0 to HOMO-1 and LUMO-1 to HOMO-2 transitions. Emission is less likely than absorption; therefore, the energy can be assumed to be dissipated with some molecular motion. The absorption and emission spectra show very similar peaks with a slight energy loss, which is probably due to small structural reorganizations.

In the Indigo, Selenoindigo and Tetrabromoindigo spectra, the absorption and emission intensities are similar. This situation is different from that observed in the Dichloroindigo and Thioindigo spectra, where the absorption intensity is lower than the emission intensity. This result is attributed to electrons in LUMO, during emission, are few more that which ones came from HOMO, and they contribute to this transition.

In Hemiindigo case, the emission spectra intensity is lesser than the absorption spectra; maybe for intermolecular efforts, causes by the energy absorption.

In Table 2, the differences between the absorption and emission energies are summarized, and the adsorption and emission transitions are indicated.

**Table 2:** Difference between the absorption and emission energies of each molecule

	**Transitions**	**ΔeV**
Indigo	H-0 ↔ L-0	0.08
	H-3 ↔ L-0	0.05
Dichloroindigo	H-0 ↔ L-0	0.08
	H-3 → L-0 , H-2 ← L-0	0.05
Selenoindigo	H-0 ↔ L-0	0.12
	H-4 ↔ L-0	0.09
Thioindigo	H-0 ↔ L-0	0.15
	H-3 → L-0 , H-4 ← L-0	0.1
Tetrabromoindigo	H-0 ↔ L-0	0.06
	H-2 ↔ L-0	0.01
Hemiindigo	H-1 ↔ L-0	0.07
	H-2 ↔ L-1	0.22

Absorption and emission transitions are also shown.

In this study, the energy difference (eV) between the absorption and emission transitions is used as a basis for comparison of the variation between the different molecules, in N substitution, must probably caused for the increase in the electron charge on that zone, that originated for the weightiest atoms; as such, the transitions of HOMO-0 to and from LUMO-0 can be proved to remain close if the substitutions in the Indigo molecule are made on the hydrogen atoms by Cl or Br. However, if substitutions are made on the nitrogen atom by S or Se, the difference between the transitions of HOMO-0 to and from LUMO-0 are greater and are different from those that occur in the indigo molecule.

No conclusions were reached with respect to Hemiindigo because the absorption transitions do not correspond with the emission transitions. This result is interesting and is attributable to the fact that the molecule undergoes geometric modifications, which is a process that requires a different set of experiments.

This energy differences can be using to compare the dissipation energy by photoemission; the smaller values provide a glimpse of which systems dissipate more energy by photoemission.

We believe that Tetrabromoindigo dissipates more energy by photoemission, followed closely by Indigo and Dichloroindigo. Tetrabromoindigo and Dichloroindigo are molecules with the H substitution. And the N-H substitutions are the systems with the smaller dissipation energy by photoemission.

This can be associated to geometric alteration caused by the substitutions. For H substitutions the only modification occurs in C-Br o C-Cl distances, that increase 60 % for C-Cl bond and 75 % for C-Br. In the case of N-H substitution for S, distances S-C increase 30 % in comparison to N-C distances, and angles C-S-C decrease 18 %; in the case of N-H substitution for Se, distances Se-C increase 40 % with respect to N-C distances, and angles C-Se-C decrease 20 % in comparison to C-N-C.

We considered that the geometric distortions of the five-membered ring are responsible of the differences in the observed absorption an emission spectra. The systems with the N-H substitutions dissipate less energy by photoemission, because part of the energy is dissipated through geometric distortions.

## Conclusions

Each of the molecules was theoretically analyzed. The calculations show that the adsorption and emission spectra are affected by the substitution position. When a Hydrogen atom of the molecule is substituted by Cl or Br, there is practically no variation in the difference between the adsorbed and emitted energies with respect to those observed for the Indigo molecule; however, when the Nitrogen atom is substituted by S or Se, the energy differences are greater. In addition, when N is substituted by S or Ser, the transition probabilities decrease; they increase, however, when H is substituted by Cl or Br.

## Competing interests

The authors declare that they have no competing interests.

## Authors’ contributions

FCN and DGM carried out all the calculations, and analyzed the data together. Both authors read and approved the final manuscript.
